# Post-doctoral fellow and faculty perceptions and experiences of inclusion at academic medical centers

**DOI:** 10.1017/cts.2025.21

**Published:** 2025-03-27

**Authors:** Chantele Mitchell-Miland, Doris M. Rubio, Tiffany L. Gary-Webb, Galen Switzer, Gretchen E. White, Natalia E. Morone, Audrey J. Murrell, Megan Hamm

**Affiliations:** 1 Institute for Clinical Research Education, University of Pittsburgh Schools of the Health Sciences, Pittsburgh, PA, USA; 2 Department of Epidemiology, School of Public Health, University of Pittsburgh, Pittsburgh, PA, USA; 3 Department of Medicine, Division of General Internal Medicine, Medicine, Psychiatry and Clinical and Translational Science, Pittsburgh, PA, USA; 4 Department of Medicine, Boston Medical Center, Boston University Chobanian & Avedisian School of Medicine, Boston, MA, USA; 5 College of Business Administration, University of Pittsburgh, Pittsburgh, PA, USA

**Keywords:** Inclusion, biomedical researchers, qualitative, underrepresented, workforce

## Abstract

**Introduction:**

The purpose of this research was to understand perceptions and experiences of inclusion among underrepresented early-career biomedical researchers (postdoctoral fellows and early-career faculty) enrolled in the Building Up study. Because inclusion is vital to job satisfaction and engagement, our goal was to shed light on aspects of and barriers to inclusion within the academic workforce.

**Methods:**

We used qualitative interviews to assess workplace experiences of 25 underrepresented postdoctoral fellows and early-career faculty including: their daily work experiences; sense of the workplace culture within the institutions; experiences with microaggressions, racism, and discrimination; and whether the diversity, equity, and inclusion (DEI) policies and practices at their institution enhanced their experiences. Using qualitative methods, we identified themes that highlighted high-level characteristics of inclusion.

**Results:**

Four distinct themes were identified: (1) participants appreciated the flexibility, versatility, and sense of fulfillment of their positions which enhanced feelings of inclusion; (2) greater psychological safety led to a greater sense of belonging to a research community; (3) participants had varied experiences of inclusion in the presence of microaggressions, racism, and discrimination; and (4) access to opportunities and resources increased feelings of value within the workplace.

**Discussion:**

Our findings provide new insight into how inclusion is experienced within the institution among underrepresented early-career biomedical researchers. This research points to specific approaches that could be used to enhance experiences of inclusion and to address barriers. More research is needed to understand how to accomplish a balance between the two, so that perceptions of inclusion outweigh negative experiences.

## Introduction

By 2050, the general US population will be less than 50% non-Hispanic White [1], yet diversity within the biomedical workforce is not reflecting these rapid changes [2,3]. Recent attention has been devoted to the need for effective strategies for diversifying the biomedical workforce [4] especially given the dual impact of systemic racism and the long-term disruption of the COVID-19 global pandemic[4,5]. The National Institutes of Health (NIH) prioritizes increasing representation in biomedical research including underrepresented racial and ethnic groups, individuals with disabilities, individuals from disadvantaged backgrounds, and women (in select biomedical research areas) [6].

Diverse biomedical workforces are important for several reasons. Research has shown that diverse research teams are more productive than more homogeneous teams and produce innovative research [7]. Additionally, diversity within the workforce offers more opportunities for creative approaches to new and existing healthcare problems [8] and advocacy for change of existing power structures that cause inequities [9].

Of particular importance to the efforts to diversify the biomedical workforce is the study of underrepresented researchers’ experiences within the workforce, which has implications for retention efforts. It is known that biomedical researchers from underrepresented racial and ethnic backgrounds (Black or African American, Hispanic or Latina/o, American Indian or Alaska Native, Native Hawaiian or other Pacific Islander) have higher attrition rates throughout their career trajectory compared to their White and Asian counterparts [10–13]. Discrimination is thought to play a large role in the high attrition rates [14,15], however, there may be other factors that are not being examined. Many research and healthcare agencies, including the NIH, are exploring factors and methods to learn more about these groups [7].

Inclusion is known to be a key concept in determining the effectiveness of recruitment and demonstrating the impact on retention for underrepresented biomedical researchers. In fact, some argue that without inclusion, diversity efforts alone may be insufficient to impact changes in biomedical and other STEM fields [16,17] because inclusion addresses factors such as belongingness, psychological safety, and work engagement, which create environments where diversity can thrive. While there is no gold standard definition of inclusion, most definitions include two main components. The first is having a sense of psychological safety or an individuals’ perceptions of favorable consequences related to interpersonal interactions within their work environment [18–20]. There are four components of psychological safety: (1) inclusion safety—members feel safe to belong to the team; (2) learner safety—members are able to learn through asking questions; (3) contributor safety—members feel safe to contribute their own ideas, without fear of embarrassment or ridicule; and (4) challenger safety—members can question others’ ideas or suggest significant changes to ideas, plans, or ways of working [20,21]. The second component is having equitable access to opportunities and resources within the organization and being considered an “insider” that contributes to the organization’s success [22]. Several studies have identified an association between feelings and perceptions of inclusion and work engagement [23]; however, more research is needed to understand inclusion among underrepresented biomedical researchers. Engagement refers to an employee’s commitment to contributing to the organization’s goal [24,25] and is related to work experience that is purposeful, fulfilling, and positive [26].

The Building Up a Diverse Biomedical Research Workforce Study (Building Up) offers a unique opportunity to understand inclusion among underrepresented early-career biomedical researchers (postdoctoral fellows and early-career faculty). Building Up is a large, national trial that tested the effectiveness of an intervention to increase research productivity, belonginess and psychological safety. In this secondary study, we used qualitative interviews to explore Building Up participants’ feelings of inclusion at their institutions.

## Methods

Participants were recruited for this secondary, qualitative study from Building Up [27], which is a two-arm cluster randomized trial at 25 academic medical institutions (23 primarily white institutions and 2 minority serving institutions) that tests the effectiveness of two intervention arms with varying intensity of 4 intervention components (monthly sessions, networking, coursework, and mentoring) to increase research productivity. For this study, a subsample of 33 participants were randomly selected from the Building Up participant pool and were emailed an invitation to participate in an interview. We sought to oversample from the two minority serving institutions (MSI), in the case that participant’s experiences were qualitatively different and to have a sample with approximately equal numbers from each intervention arm. Interviews were conducted from February 23 to June 18, 2022. If a potential participant declined or did not respond, another was randomly selected. A single Institutional Review Board at the University of Pittsburgh approved the protocol. Participants provided electronic informed consent and were informed that their responses were confidential.

We employed a qualitative description approach, in which researchers seek to describe participants’ thoughts and feelings on study topics without abstracting to the theoretical models, as is often the goal with qualitative research in the social sciences. This type of qualitative approach is common in qualitative studies in medical and health sciences contexts [28,29] and tends to produce actionable insights.

The primary investigator developed an interview guide covering domains of interest, including how the participant came into their current position, how they described their workday (what they liked/disliked about a typical day and what they wanted to change), and how they described their working unit (commitments to diversity, equity, and inclusion [DEI]; experiences of microaggressions, racism, and discrimination; and workplace culture). The guide was reviewed and piloted by members of the Building Up study.

Interviewing duties were split between the primary investigator and an interviewer from the Qualitative, Evaluation, and Stakeholder Engagement Research Services (Qual EASE) at the University of Pittsburgh. Interviews were conducted via Zoom or telephone, per the participants’ preference, and were audio recorded. The mean completion time for all interviews was 50 minutes (range 20 minutes – 1 hour 24 minutes) and mean completion times were similar for each interviewer (49 minutes and 52 minutes). Interview style was slightly different between interviewers, but coding was similar.

Verbatim transcripts of the audio recordings were produced by research assistants at Qual EASE. Following transcription, the primary investigator inductively developed a codebook based on the content of the interviews; the codebook was reviewed and approved by the qualitative methodologist and interviewer. The primary investigator then co-coded all transcripts with an experienced coder from Qual EASE and adjudicated all coding disagreements to full agreement. The completed coding served as a basis for a thematic analysis [30,31] conducted by the primary investigator. The resulting themes were shared and discussed with the rest of the study team as a form of investigator triangulation and to allow team members to provide feedback to refine the themes. Participant characteristics were presented using frequencies and percentages for categorical variables and mean and standard deviation for continuous variables.

## Results

Of the 33 people we contacted, 25 (16 faculty and 9 post-doctoral fellows) responded and were interviewed. The eight participants that did not participate either did not respond to the email (*N* = 6) or no-showed (*N* = 2) for their appointments. Of those that did respond, participants’ racial/ethnic identities were as follows: Black (36%), more than one race (28%), Asian (12%), Latino/a (12%), White (8%), and Middle Eastern (4%). Two participants (8%) reported a disability and 76% of participants were from the control arm. Compared to the main study, participants in this analysis were slightly older (36 vs. 39), had slightly more women (80% vs. 84%), were less likely to report a disability (6% vs. 8%), and more likely to hold faculty positions (64% vs. 53%) [27]. Most participants were in academic positions, although a few participants had transitioned to industry positions. The participants were working at 16 of the 25 institutions included in the larger study including one of the MSI (participants from the other MSI did not respond). For most, regardless of type of position, their primary work activities included meetings, research, teaching, administrative duties, mentoring, and supervisory roles.

Our research team identified four distinct themes: (1) Participants appreciated the flexibility, versatility, and sense of fulfillment of their positions which enhanced feelings of inclusion; (2) Greater psychological safety led to a greater sense of belonging to a research community; (3) Participants had varied experiences of inclusion in the presence of microaggressions, racism, and discrimination; and (4) Access to opportunities and resources increased feelings of value within the workplace.

### Theme 1: Participants appreciated the flexibility, versatility, and sense of fulfillment of their positions which enhanced feelings of inclusion

Many participants described working in environments that were conducive to inclusion. Most participants enjoyed the flexibility (ability to organize schedule including working remotely and “freedom” to make decisions about how projects are chosen and conducted) and versatility (use of multiple skillsets), of their positions, which they said enhanced their sense of creativity and innovation. Specifically, participants enjoyed how they could set their own schedules to align with when they felt they functioned best. They also discussed how the research they did was rewarding because it led to new discoveries and methods of addressing existing problems. Having a sense of autonomy while developing new research or running lab experiments led participants to feel productive. Being able to conduct and make progress on their projects brought a sense of fulfillment and inclusion.

Additionally, when participants’ work was in line with their overall career goals, they reported feeling fulfilment with a significant sense of purpose. This helped participants feel part of the institution’s overall mission, which is a component of inclusion. This sense of fulfillment was amplified when participants felt supported via assistance to complete their work or recognition of their efforts by their mentor and/or supervisor. Many participants also commented on how the sense of fulfilment was enhanced because they were making a difference as an underrepresented biomedical researcher.

At times, participants felt there were barriers to having the flexibility that they needed. For example, some participants discussed how having duties, such as clinical and/or teaching responsibilities, sometimes took precedence over the research that they wanted to focus on. Other times, they reported feeling undervalued when they were asked to do service work such as DEI service that was meaningful but did not contribute to their research goals. When leadership’s priorities were not in line with the participant’s, the participant did not have as much autonomy around how they spent their time. While this did not change their sense of fulfillment about their research, it often elicited feelings of conflict, frustration, and stress as they tried to accomplish all the tasks that they needed to do, which challenged their sense of inclusion. At times participants expressed uncertainty about how to navigate these situations with their superiors. In some instances, participants described their experiences as a “tax” of being underrepresented compared to their majority counterparts.

See Table 1 for representative quotes.

**Table 1.** Quotes representing theme 1 – participants appreciated the flexibility, versatility, and sense of fulfillment of their positions which enhanced feelings of inclusion

Flexibility	There’s a lot of things I like. I like having the flexibility to sort of make my schedule around when [I] do the best work. I really like the research that I’m working on. I am really inspired by the other people who are in my department who are doing similar type of work. And it feels like the research can really help like, sort of change policy and practice, which I really like. [Participant_145]
Conflicts with research time	Because I’m a clinician scientist, I’m doing a combination of clinical work as well as research, and there is—when you’re applying and trying to advance, there’s emphasis on both, but in practice there’s a lot more emphasis on the clinical responsibilities and the demands of the division. And so, my day to day basis, I may have let’s say 85% clinical requirements versus let’s say 15% research. But, you know, there’s no one actually looking at your schedule and saying, “okay you’re supposed to have 15% clinical work, so do not come in on this day” And so a lot of the pressure, and this isn’t intended pressure, it just happens. And this is why I’m saying thing don’t sort of move in concert, it just happens as a result of the demands from the clinical requirements side. And I frequently end up coming in to do procedures when I shouldn’t, I should be focused on my research. [Participant_393]

### Theme 2: Greater psychological safety led to a greater sense of belonging to a research community

Participants discussed how elements of inclusion safety or feeling safe to belong to a team, a component of psychological safety, affected their feelings of being in a research community. (Participants did not specifically use the term “inclusion safety” or “psychological safety” in their descriptions.) One example included feeling welcomed by their colleagues when joining their working unit via welcoming emails or other invitations to be part of the research team. Participants also described how collaborative and supportive their environment was. These environments were described as not being competitive but, rather, were contexts in which participants were able to discuss and receive support from their coworkers, mentors, leadership, and collaborative networks outside of their immediate workplace environments. Participants also reported that greater psychological safety allowed them to advocate for additional support they might need to conduct their research.

Participants mentioned that “strong” leadership increased their feelings of psychological safety. They described strong leaders in the following ways: leading by example, being open to listening and addressing the participant’s or others concerns, advocating for and supporting participants when needed, being receptive to the needs of all, and being approachable. Strong leadership played a role in feeling supported in their research community and often set the tone for other interactions within the work environment.

When participants reported lower trust of their colleagues, mentors, and/or supervisors, and thus lower inclusion safety, they did not feel as connected to their research community. For example, participants offered scenarios in which they were ignored during introductions to coworkers or were not spoken to by senior research faculty when in the elevator together which made them feel as though they were not accepted within the research community. At times, participants attributed being ignored to their underrepresented status and perceived that their majority counterparts were not having the same experience. Other regular interactions also made them feel like they were not part of the team. These missed interactions were perceived as important because they were seen as opportunities to network, collaborate, and generally be viewed as a colleague. Others with an international background sometimes felt that they were not taken seriously because they were not from the U.S or because English was not their first language. Participants also cited lack of diversity, generational differences, differences in how different degrees were valued, and lack of support from leadership as playing a role in strained interactions that led to lower inclusion safety. Additionally, some participants discussed how they did not know how to interact with certain individuals (particularly those with majority status) or felt uncomfortable doing so, which often led to self-induced isolation. These experiences resulted in feelings of stress and of being undervalued and sometimes contributed to individuals leaving a department or an institution.

See Table 2 for representative quotes.

**Table 2.** Quotes representing theme 2 –greater psychological safety led to a greater sense of belonging to a research community

Feeling welcomed	The department they have a really great, um, so anytime a new faculty members joins the department, there is, like, an email. A formal email that’s sent. And it was pretty nice. Somebody from the communications department reached out to me prior to my official start date and, um, asked me to review the wording of just a little announcement that was going to be sent to everybody in the department. And, um, I think that really went a long way. Um, and, yeah, it’s really nice. [Participant_349]
Collaborative and Supportive Environment	So, the core nucleus of the group has known each other for a very long time, and then they’ve integrated newer faculty members and stuff like that. It’s a very—it’s a fairly collaborative effort, so people, you know, we knock on doors when we can and you know, talk to people, and people are fairly open with what they’re doing. There’s not a whole bunch of “this is mine, and I can’t talk about it to you” and stuff like that. So, I think around there, everything’s very open, very honest. People try and be helpful. [Participant_461]
Not being welcomed	Like, you come to a place and they tell you, and they feel like saying “hey, you don’t deserve to be here,” which is part of the common dialog that we have now. Like, this insistence of people that for whatever reason or the other, someone better could have been there and they feel entitled just to say that, and you wonder if it is racially-related. Again, I—I put myself—and I asked this to many of my white folks and stuff, right, like, have you ever been told stuff like that? And they tell me no, not really. It’s kind of a little survey that you run around, and you notice that some people are like, “no, nobody would tell me that,.” Even if it was true, they would tell me [happily] “nobody would tell me anything like that.” [Participant_411]

### Theme 3: Participants had varied experiences of inclusion in the presence of microaggressions, racism, and discrimination

Participants described aspects of inclusion safety as they discussed different ways that they interpreted and experienced microaggressions, racism, and discrimination directly or indirectly which could have occurred in their work unit or within the broader institution. Often during the interview, participants would describe how they suppressed experiences of microaggressions, racism, and discrimination as a coping strategy so that they could focus on their research. For this reason, participants may not have recalled all the negative experiences they had. Additionally, because ignoring the occurrence was a way of dealing with microaggressions, racism, and discrimination, they too might have chosen not to share certain occurrences. Many participants reflected on how they were not often asked for input about these experiences within their work culture.

Participants who recalled direct experiences of microaggressions, racism, or discrimination described times when they personally experienced an interaction that called into question their ability or character in a negative stereotypical manner. Although these experiences were often reported as being stressful, participants had a variety of ways of dealing with them. Some participants reported the occurrence to their supervisor or another reporting body, some bonded with other coworkers to deal with the occurrence (this was most common when discriminatory patient behavior was involved), and others ignored the experience in favor of keeping the peace. Participants who reported having organizational support stated that their complaints were taken seriously and were more likely to report feelings of inclusion and belonging compared to those that did not report feeling supported.

Participants had different reactions to indirect experiences of microaggression, racism, and discrimination (i.e., observed or shared experiences that did not directly happen to them). Some participants were able to offer support to an individual that experienced a microaggression or instance of racism or discrimination, while other participants were not sure how to show support. Often, participants experienced stress due to indirect experiences, especially if they did not feel the situation was addressed well. Such indirect experiences sometimes caused participants to question the values and standards within the workplace, which affected their feelings of inclusion. In rare cases, participants reported leaving or wanting to leave their institution due to indirect experiences.

See Table 3 for representative quotes.

**Table 3.** Quotes representing theme 3 – participants had varied experiences of inclusion in the presence of microaggressions, racism, and discrimination

Microaggressions in a supportive environment	Yeah, I think there are always microaggressions. Uh, there’s—there are always members of the community who will say insensitive things sometimes. To be honest, I have a pretty healthy habit of staying away from people that I find not very supportive, so I’ve kind of self-selected the environment within this larger environment that I choose to engage with. But it’s not—I would say I do not think it’s pervasive. I think—I think there are examples that I can think of that are few and far between, and I think the larger culture and environment is supportive. [Participant_393]
Unaddressed Microaggressions	I guess I just have felt once in one meeting, right, they were talking about… I do not know, people of color, I don’t even know even [what for], and I didn’t even take it personally until the person that was making the comment said, “oh I didn’t mean it for you.” And I was like—I really, only after she said that … And then I start wondering myself, how many times has this happened? And actually mean it, right? Like in retrospective. But not necessarily. [Participant_714]
Indirect experiences	Um, yes, but not because I am white. I like fully—nobody has said things to me personally, but there is an admin in our Institute and for some reason she—like, nobody told her like [laugh] you cannot say those things, because when I started, she was like coming to my office routinely and saying incredibly racist, biased things. And I was junior—I still am—I was like, I have no idea how I’m supposed to handle this. [Participant_717]

### Theme 4: Access to opportunities and resources increased feelings of value within the workplace

Access to opportunities and resources were important for inclusion. Participants described how access to opportunities, including opportunities to communicate their needs, increased their feelings of being valued, which is related to contributor safety or feeling safe to share ideas. Participants described that sharing needs as an underrepresented researcher felt vulnerable because it could be interpreted as not being qualified for their job. Participants appreciated opportunities to discuss their research plans with other coworkers, or to have a wide range of individuals on their mentoring and collaborative teams, especially those from underrepresented groups. In some situations, participants commented on how not being able to work with mentors from underrepresented groups was not as important to them because they felt supported by their existing mentors. Participants greatly valued moments when they asked for things that they needed and their needs were taken seriously—particularly if they felt vulnerable when asking.

Regarding career advancement, participants were very appreciative when they were presented with opportunities that would contribute to their career growth. This largely included being able to share their research progress with the scientific community, being able to collaborate with other researchers, or having opportunities to develop skills that would enhance their work. While some participants did mention opportunities to do service work, such as creating DEI curriculum, they often mentioned that these did not count toward their advancement and took a considerable amount of time. While many discussed enjoying these types of activities, they also wanted to be considered for opportunities that would enhance advancement at their institutions (i.e. publications and grants).

Regarding resources, participants valued having access to resources (e.g., competitive pay, access to labs and lab equipment, startup packages, trainings, and personnel) that guided them as they were developing their research careers. These resources either lessened the amount of work the participant had and/or allowed them to be more effective and efficient. Others who did not have access to these resources mentioned that having them would make a significant difference. Generally, having access to these resources increased the participant’s feeling of value in the workplace because they felt that leadership was investing in them, and they had the tools to be more effective at their jobs.

Participants specifically described the importance of improving the effectiveness of DEI policies and practices, as resources, leading to climate and culture change, ensuring that the participant’s unique needs are met. Participants mentioned that effective practices and policies reflect and in fact drive actual—as opposed to nominal—culture changes within the workplace. Participants expressed how these culture shifts facilitate communication of unique challenges faced by early career researchers, especially those with underrepresented status. When participants did not feel genuine intentions behind their institution’s DEI policies, they felt less inclusion and did not feel as valued in their workplace.

See Table 4 for representative quotes.

**Table 4.** Quotes representing theme 4 –access to opportunities and resources increased feelings of value within the workplace

Opportunity to Communicate Needs	…I was just coming from my maternity leave, one of the initial conversations that I had with the PI was that I needed some time to—I needed him to be patient and I needed some time as well to learn how to manage, you know, this new step in my life, this new phase in my life in which I have to care for a little thing. [laugh] And I said that with, you know, a lot of fear, actually, of what the response would be, and he just looked at me and said, “I wouldn’t expect anything different.” And so, that was very important to me…[Participant_526]
Collaborative Resources and Support through Strong leadership	Yeah, I like that it is collaborative. I like that it is constantly, you know, different. I love that there’s incredible support from the department chair- down, but we don’t feel like we’re down or below or beneath anybody, It’s very, very collaborative, very supportive. The department chair oversees the largest department and the highest number of divisions in the institution, and is such a great leader that he remembers details about people, he genuinely cares. He remembers your projects, your personal stuff, so that’s a really nice—really nice thing. I think that doesn’t probably exist in a lot of places. [Participant_35]

PI: Principal Investigator.

## Discussion

We identified four main themes that improved inclusion at the institutional level among Building Up participants. The themes illustrate how inclusion is enhanced when there is a sense of flexibility and versatility around the work that is being done. Inclusion is further enhanced when the participants felt psychological safety within their research community; felt supported in the presence of microaggressions, racism, and discrimination; and felt that the general environment was supportive in terms of opportunities and resources. However, our findings suggest nuanced or subtle differences in the ways that participants perceived inclusion within their work environments. For example, if a participant felt supported within their work environment, experiences of racism, microaggressions, or discrimination may not have been as detrimental to their sense of inclusion as those participants who did not feel supported. Additionally, participants expressed differences in how they navigated issues at work. For example, some participants focused on finding supportive groups in the face of situations that seemed hostile due to discrimination while others were bothered by hostility.

These findings highlight that underrepresented biomedical researchers, especially those who are early-career, perceive differences between what they need, compared to their majority counterparts, to reach the same level of psychological safety in their workplace. To elaborate, underrepresented participants expressed a desire to have their unique challenges recognized by others. Many indicated their underrepresented status meant that they needed additional resources to function equivalently to their well-represented counterparts. For example, some participants isolated because they were not sure how to interact with their well-represented counterparts or who to seek help from. While we did not include well-represented early-career biomedical researchers in this research, several studies have indicated that they do not report the same level of adversity as their underrepresented counterparts [32]. In addition, our findings suggest that psychological safety can be inhibited by missed opportunities to interact with colleagues and senior faculty. It is important to understand how and under what circumstances these missed opportunities impact inclusion and what counteractions may be employed to mitigate these impacts.

While these findings are consistent with other published literature, this study provides more context around how unconscious bias can affect inclusion. Much of the literature focuses on unconscious bias and the presence of discrimination as independent barriers to inclusion [17,33–35]. That is, an individual from an underrepresented background will not be able to feel included where unconscious bias or discrimination is present. While it is not preferable to have unconscious bias and discrimination, ridding our institutions of unconscious bias that leads to discrimination is a gargantuan task that will take massive amounts of resources and time [36,37]. Our data suggest that there are ways to improve inclusion among underrepresented biomedical researchers even when discrimination is present. Developing more robust interventions that specifically increase psychological safety, irrespective to when discrimination is present, and enhance the availability of opportunities and resources that strengthen feelings of value and belongingness is indicated.

Our study also provides more context around the relationship between psychological safety and the policies and practices of a workplace. Policies and practices are important components of psychological safety. However, our findings indicate that the presence of policies and practices alone did not increase psychological safety or feelings of inclusion. Instead, participants indicated that it was important that policies and practices were in line with the daily operations of the workplace and that they reflected the values of the members of the workplace. Thus, it is important for leaders and administrators to assess the climate of their workplace and to develop policies and practices with realistic plans for implementation and with genuine expectations and timelines [38]. For example, creating policies that endorse diversifying the workforce when 80% of the employees do not believe there is a problem with diversity is ill advised as that does not match the climate of the workplace. These policies will likely feel inauthentic to people from underrepresented backgrounds, may cultivate feelings of mistrust, and will not be executable in a reasonable time frame.

Anti-DEI legislation will likely have a significant impact on the opportunities and protections that are established and supported within institutions from recruitment to retention [39–41]. The erosion of DEI policies may have negative effects on hiring personnel that establish and reinforce DEI policies, establishing programs that focus on improving outcomes for marginalized populations (such as Building Up), and providing protections for minoritized groups [40]. It is possible that these changes will cause fear and uncertainty about the best ways to continue DEI-focused work [40]. Additionally, without specific policies and programs awareness of the unique needs of underrepresented researchers may be greatly diminished leading to more barriers and less feelings of inclusion [39]. However, as participants alluded to, there are many things that can be done in the workplace to support underrepresented researchers beyond policies. For example, leadership can be intentional about modeling equitable practices within the workplace and holding others accountable for doing the same. In line with this example, Cox and Nguyen suggest movement towards accountability practices in the face of threats to DEI polices [41]. While DEI policies and programs focus on the means to achieve diversity goal process (e.g., anti-bias training), accountability practices focus on accountability to the goal itself (e.g., equitable career advancement). Institutions and workplaces will likely still be able to implement accountability practices that monitor and assess goal achievement and promote belonging and inclusion without violating laws [40,41]. Accountability structures and practices have been associated with inclusion [42] and career advancement of underrepresented individuals [41].

This study has both strengths and limitations. As for its strengths, we assessed perceptions of inclusion among a diverse group of early-career biomedical researchers from backgrounds underrepresented in science. Additionally, the study examined experiences of underrepresented individuals using the NIH definition of underrepresented. Even though we were not able to report on differences between specific groups (e.g., by race, gender identity, sexuality), we believe that the data is robust enough to provide transferable insight into underrepresented individuals’ experiences. Future research should include within-group differences, which may reveal differences in experiences of inclusion. For example, gender differences in personality traits might influence how individuals from different genders perceive inclusion [43]. Understanding the circumstances in which underrepresented early-career biomedical researchers feel included can lead to more effective policy and practice changes that may influence retention. Another strength is that we sampled from a large research study of early-career biomedical researchers and that we interviewed participants at various institutions throughout the United States. While there were differences in the positions and resources of participants at each institution, some clear commonalities appeared regarding experiences of inclusion.

Our study included some limitations. All interviews used participants’ accounts of their experiences in the workplace with the majority of participants coming from the control study arm. Participants may have made errors in the order in which they remembered their experiences, or they may have not recalled specific aspects of their experiences and the participant’s study arm may have influenced their participation or responses. However, participants were asked to base their feelings of inclusion on their experiences within their work unit and institution and were not asked to discuss their experiences in the study. Because psychological safety and inclusion are largely based on perception, we do not believe these errors affected our study findings. Participants may have also under-reported negative experiences due to concern over self-perception or the possible impact it might have had to their institution if they were identified. This study also only evaluated experiences and perceptions of inclusion among academic centers. Biomedical research done in other settings, such as industry, may have other defining factors. Lastly, while we sought to be inclusive of all 25 institutions included in the larger Building Up study, our sample included participants who were available and self-selected to be interviewed. This may have introduced bias in the sampling process.

Even given these limitations, our findings have several implications that indicate various future research directions. Our findings suggest several areas of intervention that may improve how individuals experience inclusion, including thoughtfully implementing policies and practices, providing additional focused resources for people from underrepresented backgrounds, enhancing opportunities for communication and collaboration, and tailoring ways to engage leadership to provide and model effective ways to interact within the workplace. These findings also offer new lines of inquiry. For example, different gender specific personality characteristics (i.e., women are often found to be more agreeable than men) may have impacts on their experiences of inclusion. By better understanding how nuance may affect implementation strategies, there may be better ways to improve feelings of inclusion within academic medical centers which can improve belongingness, psychological safety, and work engagement which are related to retention efforts.

## Data Availability

The datasets [GENERATED/ANALYZED] for this study can be found in the [NAME OF REPOSITORY] [LINK]. Please see the “Availability of data” section of Materials and data policies in the Author guidelines for more details.
